# Transcriptomic analysis of the honey bee (*Apis mellifera*) queen brain reveals that gene expression is affected by pesticide exposure during development

**DOI:** 10.1371/journal.pone.0284929

**Published:** 2023-04-27

**Authors:** Myra Dickey, Elizabeth M. Walsh, Tonya F. Shepherd, Raul F. Medina, Aaron Tarone, Juliana Rangel

**Affiliations:** 1 Department of Entomology, Texas A&M University, College Station, Texas, United States of America; 2 Honey Bee Breeding, Genetics, and Physiology Research Laboratory, USDA-ARS, Baton Rouge, Louisiana, United States of America; University of Alberta, CANADA

## Abstract

Honey bees (*Apis mellifera*) play a pivotal role in agricultural production worldwide, primarily through the provision of pollination services. But despite their importance, honey bee health continues to be threatened by many factors, including parasitization by the mite *Varroa destructor*, poor queen quality, and pesticide exposure. Accumulation of pesticides in the hive’s comb matrix over time inevitably leads to the exposure of developing brood, including queens, to wax contaminated with multiple compounds. Here, we characterized the brain transcriptome of queens that were reared in wax contaminated with pesticides commonly found in commercial beekeeping operations including either (a) a combination of 204,000 ppb of *tau*-fluvalinate and 91,900 ppb of coumaphos (“FC” group), (b) a combination of 9,800 ppb of chlorpyrifos and 53,700 ppb of chlorothalonil (“CC” group), or (c) 43,000 ppb of amitraz (“A” group). Control queens were reared in pesticide-free wax. Adult queens were allowed to mate naturally before being dissected. RNA isolated from brain tissue from three individuals per treatment group was sequenced using three technical replicates per queen. Using a cutoff log_2_ fold-change value of 1.5, we identified 247 differentially expressed genes (DEGs) in the FC group, 244 in the CC treatment group, and 668 in the A group, when comparing each group to the control. This is the first study to examine the sublethal effects of pesticides commonly found in wax (particularly amitraz) on the queen’s brain transcriptome. Future studies should further explore the relationship between our molecular findings and the queen’s behavior and physiology.

## 1. Introduction

Wild and managed bees are constantly exposed to biotic and environmental stressors that can threaten their health [1–5]. In the last few decades, managed honey bees (*Apis mellifera*), which contribute over $200 billion to the global economy annually [6], have been subjected to several concomitant stressors that compromise their health and fitness [7, 8]. Top stressors include poor queen quality, pesticide exposure, parasites, pathogens, and malnutrition [9–14]. Of these, the most devastating threat to honey bees worldwide continues to be the ectoparasitic mite *Varroa destructor*, which feeds on the fat bodies and hemolymph of developing and adult bees, all while serving as a highly effective mechanical and biological vector of several honey bee-associated viruses [15–18]. Colonies with high *Varroa* infestations are likely to collapse and die within two years if no management action is taken against the mites [7, 11, 12, 19, 20]. Given the serious health problems caused by *Varroa*, most beekeepers use chemical products to treat colonies when levels go above the established threshold of two-to-four mites per 100 adult bees [21, 22]. However, most of these pesticides cause sublethal effects to honey bees either individually or in synergy with one another [11, 23–27].

Over the past decade, multiple surveys across the U.S. have detected high concentrations of miticides and other pesticides inside most hives, independent of the type of beekeeping management style practiced [9, 11, 24, 28]. The most comprehensive survey of pesticide residues in beekeeping operations to date was that conducted by Mullin et al. [9], who screened for the presence of over 170 chemicals and toxic metabolites in 887 bee, wax, and honey samples collected from commercial apiaries across the U.S. The authors found that 98.4% of the 259 wax samples analyzed had detectable levels of multiple pesticides. Of those, 83% contained the beekeeper-applied miticides *tau*-fluvalinate and coumaphos [9]. Even though *Varroa* developed resistance to both products by the year 2000 [29–32], they have persisted in the wax comb of many operations either because beekeepers reuse contaminated comb, or because they continue to use these products, or both [9, 11, 24, 33]. Furthermore, the miticide amitraz and its metabolites N-(2,4-dimethylphenyl)-N-methylformamidine (DMPF) and 2,4-dimethylaniline (DMA) were present in 60.5% of the wax samples analyzed. Interestingly, a recent survey documented that some mite populations in the U.S. are now resistant to amitraz [34]. While amitraz and its metabolites are legally labeled for use in agroecosystems, the U.S. Geological Survey estimated the combined application rate of these products at less than 10,000 lbs. per year [35], suggesting that beeswax contamination with those miticides (at least in the past decade) has likely been caused by beekeepers using those products for *Varroa* control instead of farmers using them intensely for protection of bee-visited crops.

Two widely used agrochemicals, the fungicide chlorothalonil and the insecticide chlorpyrifos, are also widely present in commercial beeswax [9, 11]. In the U.S., chlorothalonil is applied to crops at rates of 9 to 12 million pounds annually, while chlorpyrifos, an organophosphate, is applied at a rate of about 4 to 7 million pounds per year [36]. These two products were found in 49.2% and 63.2% of all wax samples analyzed by Mullin et al. [9], respectively, and were likely introduced into the colony by forager bees that visited flowers neighboring or within crops that had been treated with products containing those compounds. Thus, regardless of the source or route of exposure, honey bees are constantly exposed to pesticides inside the hive.

Most studies exploring the effects of pesticide contamination on bee health have focused on contact or oral exposure of larvae or adults [37–48], rather than on the effects of wax contaminated with pesticides on developing bees. Wu et al. [49] examined frames collected from commercial beekeeping operations and found that, on average, every frame was contaminated with at least ten pesticides. Of these, *tau*-fluvalinate and coumaphos were present in all the frames analyzed. As a result, brood frames contaminated with both products showed increased worker mortality and delayed development. This is not surprising, given that *tau*-fluvalinate and coumaphos stay in the wax matrix over time, and cross-contamination among combs and between colonies is possible [28]. Wax contaminated with these miticides has also been linked to “queen events,” which are described as occurrences of premature queen replacement or death [11].

Queen mortality is considered one of the leading causes of colony deaths in the U.S. [7, 12, 50–52] and is likely caused, at least in part, by queens being exposed to contaminated wax during development [28]. Not surprisingly, miticide exposure has been shown to affect queen fertility in various ways: mated queens exhibit lower count and viability of the sperm stored in their spermatheca, and young queens have lower body and ovary weight when they are exposed to miticides either orally or topically [53–56]. Previous work in our laboratory has also shown that rearing queens in wax containing field-relevant concentrations and combinations of these pesticides directly affects several aspects of queen physiology and worker behavior [25, 26, 56]. In particular, queens reared in cups coated with wax containing *tau*-fluvalinate, coumaphos, or amitraz, exhibited lower sperm count and decreased viability in their spermathecae after mating (Rangel and Tarpy 2015), showed elevated mating frequencies [25], attracted significantly fewer workers around their retinue [26], laid fewer eggs per unit time [26], and had significantly different mandibular gland chemical profiles [26], compared to queens reared in pesticide-free wax.

The behavioral and physiological changes observed in mated queens that are caused by miticide exposure during development could be linked to gene expression changes in the brain. In the model insect species *Drosophila melanogaster*, much of the wiring of the adult brain is specified during development by a genetically determined program [57]. Likewise, Vleurinck et al. [58] observed sets of differentially expressed genes (“DEGs”) involved in the specification of the brain transcriptome of honey bee workers, queens, and drones during the pupal stage. Given that pupation is a critical time point in a bee’s development [59], environmental stressors (including exposure to pesticides) could likely affect gene expression during the pupal stage. While several studies have examined the effects on the worker brain’s transcriptome after exposure to pesticides [60–62] or pathogens [63], few have focused on analyzing the transcriptome of the queen’s brain. The brain plays a vital role in inducing gene expression changes in mated queens that lead to ovary activation, egg laying, and pheromone production [64–66]. However, the few available transcriptomic studies performed on queens have only focused on gene expression differences caused by mating [64, 65, 67–69].

Due to the queen’s importance as the reproductive head of a colony, it is critical to understand how pesticide exposure during development affects her reproductive health. After observing significant changes in physiology and behavior when mated queens were reared in pesticide-laden wax [25, 26, 56], we hypothesized that such exposure would also be linked to gene expression changes in the queens’ brains. To answer this question, we reared queens in wax containing field-relevant concentrations of the same pesticide groupings as those used by Walsh et al. [25]. Emerged queens were allowed to mate naturally and their brains were then dissected for RNA extraction and transcriptomic analysis. We focused our analysis on DEGs that had predictive functions related to queen physiology and behavior. Our results indicate that rearing queens in pesticide-laden wax causes significant changes in the queen’s brain transcriptome, which could lead to downstream changes in queen reproductive output, and ultimately, colony productivity.

## 2. Materials and methods

### 2.1. Source of bees and rearing conditions

The honey bee colonies used in this study were kept at the Janice and John G. Thomas Honey Bee Facility on the RELLIS campus of Texas A&M University, Bryan, TX. All colonies were headed by sister queens of Italian descent obtained from a commercial queen producer (Olivarez Honey Queens Inc., Orland, CA) in the summer of 2017. Experimental queens were half-sisters raised from first-instar worker larvae obtained from a single source colony and were transferred into plastic cups using a common queen-rearing method known as “grafting” [70]. Each cup had previously been coated with about 200 mg of molten beeswax (certified, pesticide-free wax pellets, Koster Keunen Inc., Watertown, CT) that was kept untreated (control group) or mixed separately with either (a) a combination of 204,000 ppb of *tau*-fluvalinate and 91,900 ppb of coumaphos (>98% purity, Thermo Fisher S-980 and 50-739-91), (b) a combination of 9,800 ppb of chlorpyrifos and 53,700 ppb of chlorothalonil (>98% purity, Thermo Fisher S965A and S-915), or (c) 43,000 ppb of amitraz (>98% purity, Sigma-Aldrich 33089-61-1), as done previously by Walsh et al. [25]. These pesticides and concentrations were chosen based on their prevalence and concentrations in wax samples collected from commercial apiaries across North America [9]. While we chose the highest concentrations found in the field to detect the “worst-case” field-relevant scenarios, those concentrations can still be found in wax samples today [11, 24, 71].

The cups with grafted larvae were placed into queenless units of workers known as “cell builders” [71], inside which approximately 2,000 nurse bees cared for individual queens during development. The cell builders were not previously tested for pesticide contamination but had frames which were all less than two years old, had been kept in a non-agricultural area, and had never been treated with miticides. Two days before the queens were expected to emerge, each capped cell was caged. Emerging queens were put caged into a queenless five-frame mating nucleus colony (or mating “nuc”) composed of about 1,000 workers, two frames containing brood, one frame containing nectar and pollen, one empty frame, and one frame feeder with sugar syrup. After a two-day introduction period, the queens were marked, released from their cages, and allowed to mate naturally. Successful mating was verified by the presence or absence of worker larvae ten to fifteen days after each queen was released into her mating nuc. Once mating status was confirmed and the queens had been laying a minimum of four weeks, the queens were sacrificed and maintained at -80°C until dissection. Other tissues from the same queens, including the mandibular glands, the spermathecae, and the ovaries, were used for three separate but related studies [25, 26, 72].

### 2.2. Sample preparation, RNA extractions and cDNA library synthesis

Each queen’s brain was dissected on a bed of ice and placed in a microcentrifuge tube that was stored in a -80°C freezer (Bio-Rad Laboratories, Inc., Hercules, CA.) until RNA extraction. All equipment and dissection tools were treated previously with RNAase Away. We isolated total RNA from three biological replicates for each treatment group. Samples were placed in enough Ribozol to reach a 0.1 mL final volume. Ribozol-treated samples were homogenized using a BenchTop homogenizer (VWR catalog number 47747–307, Radnor, PA). RNA from each sample was extracted using the protocol for Ribozol^TM^ and cleaned with chloroform. After adding 1 μL of 20 μg/μL RNAase-free glycogen, RNA was precipitated overnight at -20°C. RNA pellets were generated by adding one volume of isopropanol followed by 30 min of centrifugation at 10,000 RPM at 4°C. The pellets were washed with 75% ethanol, air dried for 10 min, and resuspended in 25 μL of nuclease-free water. RNA concentrations were measured in a Qubit® 2.0 fluorometer with a Qubit® RNA HS Assay Kit (Life Technologies Corporation, Grand Island, NY). Total RNA samples were sent to the University of Texas Genomics Center and Sequencing Facility (UT GSAF) for quality assessment, cDNA library construction, and sequencing.

### 2.3. RNA sequencing and bioinformatic analyses for differentially expressed genes

Libraries, in equimolar concentrations, were sequenced on an Illumina NextSeq 500 platform with a high-output 75 paired-end sequencing (2 x 75) run using the manufacturer’s supplied custom sequencing. The quality of the data was evaluated using the FastQC software [73]. From this analysis we filtered out unqualified reads from the data, removing all adapter reads, unpaired reads, and low-quality reads using Trimmomatic [74]. The STAR software package was used for sequence alignment [75]. The reference *A*. *mellifera* genome file for alignment GCF_000002195.4_Amel_4.5_genomic.fa. HTSeq [76] was used to generate raw read counts for each gene using an intersection-nonempty parameter to account for ambiguous read mappings. We then used the EdgeR package in the R software version 3.5.1 [77] for comparative analysis of differences between treatment groups. The screening criteria for DEGs were |log_2_FC| 1.5 ≥ and *p*-value ≤ 0.01, where log_2_FC > 1.5 referred to up-regulated DEGs, and log_2_FC < -1.5 referred to down-regulated DEGs. The gene list was uploaded to the database for annotation, visualization, and integrated discovery (D.A.V.I.D.) platform [78] and referenced against the in-house database for *A*. *mellifera* on BeeBase [79]. The “Function Annotation Clustering” under “lowest” classification stringency was used to study the gene list.. To reduce the chance of encountering the false discovery of DEGs, we reported all terms with a false discovery rate (FDR) cutoff values ≤ 0.05 so that they were filtered through both, *p*- and fold-change cutoff values.

### 2.4. Confirmation of differentially expressed genes by RT-qPCR

From the RNA sequencing data, five mRNA targets were chosen to be analyzed for real-time quantitative polymerase chain reaction (RT-qPCR) that were specific to the amitraz treatment group (Table 1). We decided to focus on the amitraz group only because it yielded the highest number of DEGs and is the primary miticide used to treat against *Varroa* today, while *tau*-fluvalinate and coumaphos are no longer used in the U.S. The mRNA chosen for normalizing the data was AF441189 (*Apis mellifera* ribosomal protein 49) because of its high and invariant expression across samples in the RNA sequencing data, and because it had previously been validated as a reference housekeeping gene for expression studies in the honey bee brain [80].

**Table 1:** The top ten and bottom six genes that were differentially expressed in brain tissue from queens reared in wax contaminated with a mix of *tau*-fluvalinate and coumaphos ("FC") compared to those reared in untreated wax (control group), ranked by their log_2_ fold change (LFC). "FDR" is the false discovery rate.

BeeBase gene ID	Protein description	LFC	*p*-value	FDR
***Top ten up-regulated genes in brains from queens reared in FC-laden wax vs*. *those in the control group***				
GB44079	menin	8.96	4.50E-06	1.36E-04
GB52203	cuticular protein 13(CPR13)	8.41	1.55E-11	8.27E-09
GB42672	short-chain dehydrogenase/reductase family 16C member 6	8.01	3.88E-11	1.49E-08
GB45473	unknown	7.94	6.04E-07	2.73E-05
GB53857	metabotropic glutamate receptor B(Glurb)	7.66	1.33E-08	1.94E-06
GB42785	spermatogenesis-defective protein 39 homolog	7.51	2.11E-10	6.49E-08
GB41288	zinc finger protein 423-like	7.45	1.14E-08	1.76E-06
GB42448	double-stranded RNA-specific editase 1-like	7.01	1.14E-08	1.76E-06
GB47207	unknown	6.90	5.36E-08	4.58E-06
GB46372	unknown	6.72	4.60E-07	2.32E-05
***Top six down-regulated genes in brains from queens reared in FC-laden wax vs*. *those in the control group***				
GB40035	unknown	-4.39	1.25E-06	4.71E-05
GB53516	putative fatty acyl-CoA reductase	-1.87	3.51E-08	3.37E-06
GB42892	uncharacterized	-1.74	1.42E-04	2.48E-03
GB45626	unknown	-1.70	1.40E-06	5.13E-05
GB53408	unknown	-1.66	6.71E-04	8.71E-03
GB41608	uncharacterized	-1.64	6.85E-07	2.93E-05

Primers were designed to span exon junctions and to detect all isoforms of each gene product of interest using the Primer-BLAST software (NCBI). Primer sequences are listed in Table 1. Using the same source material as that for RNA sequencing, cDNA was synthesized from 100 ng of RNA. The total RNA was treated with DNase and reverse transcribed using an iScript^TM^ gDNA Clear cDNA Synthesis kit (Bio-Rad Laboratories, Inc.). Synthesized cDNA was diluted ten-fold and 1 μL of that dilution was used in a 10-μL qPCR reaction. Diluted cDNA acted as a template for all qPCR reactions. All amplifications used Power SYBR^TM^ Green (Thermo Fisher Scientific, Waltham, MA) in triplicate reactions (10 μL each) with primers at a final concentration of 100 nM. Standard cycling conditions (50°C for 2 min, 95°C for 2 min, then 40 cycles of 95°C for 15 s, and 60°C for 1 min) and melt curve analyses (65°C to 95°C in 0.5°C increments every 5 s) were used on a CFXTM Real-Time system (Bio-Rad Laboratories, Inc.). All qPCR analyses were done using the CFX Manager 3.1 software. Amplification efficiencies were calculated and used for correction in all normalized fold expression analyses, performed by the CFXTM Manager 3.1 software. Relative quantification was analyzed by means of the comparative C_T_ (∆∆CT) method, as done previously [81].

### 2.5. Statistical analysis

For the RNA sequencing data, we used the negative binomial distribution analysis on the edgeR program on version 3.5.1 of R to estimate means and variances for each gene per sample based on the cDNA library size. We then performed Fisher’s exact tests to calculate *p*-values. If a *p*-value was < 0.01 when comparing the relative expression of a gene between the control group and any given treatment group, then that gene was considered to be differentially expressed for that comparison.

## 3. Results

### 3.1. RNA sequencing of honey bee brain tissue

We isolated between 1 μg and 2 μg of purified RNA from each mated queen brain tissue. RNA sequencing data were generated from queens belonging to one of four treatment groups: 1) queens reared in untreated wax (control, or “C” group); or queens reared in wax containing either 2) a mix of *tau*-fluvalinate and coumaphos (“FC” group), 3) a mix of chlorothalonil and chlorpyrifos (“CC” group), or 4) amitraz only (“A” group). We created four technical replicate cDNA libraries for each of the three biological replicates per each of the four treatment groups, for a total of 48 cDNA libraries. RNA sequencing of all libraries resulted in 814 billion reads. Of those, 219 billion reads were filtered out. The remaining 595 billion reads were then mapped to the *A*. *mellifera* genome. The number of total reads and mapped reads are listed in S1 Table for each of the 48 libraries. The entire datasets for each cDNA library are available at NCBI (Bioproject # PRJNA575879).

Our RNA sequencing data revealed 247 DEGs in brain tissue from queens reared in wax containing *tau*-fluvalinate and coumaphos compared to those in the control group. Of those, six were down-regulated and 241 were up-regulated (S2 Table). The ten highest and ten lowest expressed genes in this comparison are listed in Table 1. A total of 244 DEGs were found in brain tissue from queens reared in wax containing chlorothalonil and chlorpyrifos compared to the control group. Of those, six were down-regulated and 238 were up-regulated (S3 Table). Table 2 lists the ten most up-regulated and ten most down-regulated genes in that comparison. Finally, 668 genes were differentially expressed in brain tissue from queens reared in amitraz-laden wax compared to those in the control group. Of those, 617 were down-regulated, while 49 were up-regulated (S4 Table). The ten highest and ten lowest expressed genes in that comparison are listed in Table 3.

**Table 2:** The top ten and bottom six genes that were differentially expressed in brain tissue from queens reared in wax contaminated with a mix of chlorpyrifos and chlorothalonil ("CC") compared to those reared in untreated wax (control group), ranked by their log_2_ fold change (LFC). "FDR" is the false discovery rate.

BeeBase gene ID	Protein description	LFC	*P*-value	FDR
***Top ten up-regulated genes in brains from queens reared in CC-laden wax vs*. *those in the control group***				
GB44079	menin	9.84	1.68E-06	7.06E-05
GB52203	chymotrypsin inhibitor	8.69	8.72E-12	6.71E-09
GB53857	metabotropic glutamate receptor B(Glurb)	7.79	9.54E-09	1.73E-06
GB42785	spermatogenesis-defective protein 39 homolog	7.72	1.28E-10	6.55E-08
GB44220	titin homolog	6.92	4.79E-12	4.91E-09
GB40848	dentin sialophosphoprotein-like	6.79	8.47E-13	2.61E-09
GB47216	unknown	6.16	3.81E-08	4.64E-06
GB41288	zinc finger protein 423-like	6.09	1.71E-07	1.29E-05
GB47207	unknown	5.98	1.32E-07	1.13E-05
GB43882	alpha-mannosidase 2	5.89	2.26E-12	3.47E-09
***Top six down-regulated genes in brains from queens reared in CC-laden wax vs*. *those in the control group***				
GB50121	chymotrypsin inhibitor	-4.19	1.42E-07	1.16E-05
GB40035	unknown	-2.81	2.80E-03	2.84E-02
GB50187	unknown	-2.26	2.43E-06	9.22E-05
GB42052	short-chain dehydrogenase/reductase	-2.14	6.84E-05	1.34E-03
GB53852	paired amphipathic helix protein Sin3a	-1.66	6.51E-04	8.28E-03
GB42551	alpha-N-acetylglucosaminidase	-1.53	5.46E-05	1.13E-03

**Table 3:** The top ten and bottom ten genes that were differentially expressed in brain tissue from queens reared in amitraz-laden wax compared to those reared in untreated wax (control group), ranked by their log_2_ fold change (LFC). "FDR" is the false discovery rate.

BeeBase gene ID	Protein description	LFC	*P*-value	FDR
***Top ten up-regulated genes in brains from queens reared in amitraz-laden wax vs*. *those in the control group***				
GB52328	protein patched	4.14	2.89E-06	1.61E-05
GB55498	microphthalmia-associated transcription factor	3.99	6.95E-07	4.27E-06
GB53519	unknown	3.65	5.18E-08	3.89E-07
GB44978	chitinase domain-containing protein 1	3.14	5.72E-06	3.00E-05
GB42058	helicase SKI2W	2.91	8.25E-07	5.00E-06
GB47551	putative mediator of RNA polymerase II transcription subunit 26	2.81	3.47E-06	1.90E-05
GB51075	odorant receptor Or1-like	2.64	1.94E-06	1.12E-05
GB42785	spermatogenesis-defective protein 39 homolog	2.61	7.47E-04	2.70E-03
GB41456	unknown	2.55	6.43E-07	4.01E-06
GB50057	dihydroorotate dehydrogenase (quinone), mitochondrial	2.42	3.00E-10	3.66E-09
***Top ten down-regulated genes in brains from queens reared in amitraz-laden wax vs*. *those in the control group***				
GB46164	unknown	-8.16	4.33E-17	2.22E-14
GB41855	patched domain-containing protein 3	-7.53	1.03E-16	4.51E-14
GB49752	uncharacterized	-7.00	6.61E-13	2.73E-11
GB54646	unknown	-6.90	3.48E-16	1.06E-13
GB49700	facilitated trehalose transporter Tret1-like	-6.62	5.78E-10	6.55E-09
GB47241	unknown	-6.62	1.22E-12	4.37E-11
GB50368	unknown	-6.53	1.51E-14	1.60E-12
GB48064	28S ribosomal protein S15, mitochondrial	-6.50	4.75E-16	1.12E-13
GB47623	uncharacterized	-6.34	1.06E-13	6.91E-12
GB45473	unknown	-6.33	9.52E-08	6.81E-07

**Fig 1:**
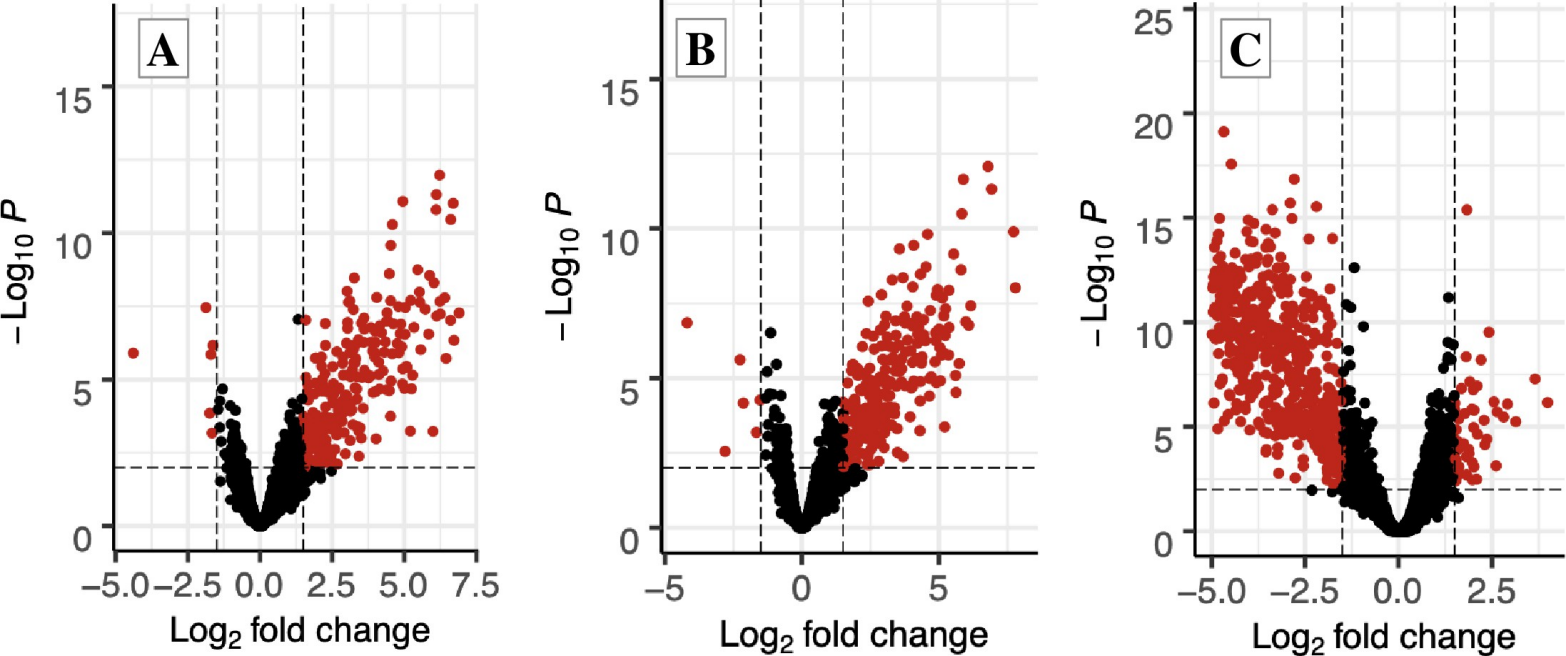
Volcano plots for gene expression in the brain tissue of mated honey bee queens that were reared in beeswax that was either kept untreated (control group) or mixed with field-relevant concentrations of miticides. The volcano plots show differentially expressed genes (red dots) when comparing gene expression among a) control queens and those reared in wax containing *tau*-fluvalinate and coumaphos, b) control queens and queens reared in wax mixed with chlorothalonil and chlorpyrifos, or c) control queens and queens reared in amitraz-treated wax. Each dot represents one gene. Red dots represent genes that only met the fold-change cut off (|log2 (fold change)| ≥ 1.5). Blue dots represent genes that only met the p-value cutoff (*p* > 0.01). Grey dots represent genes that were not differentially expressed between paired groups (*p* < 0.01 and |log2 (fold change)| ≥ 1.5).

Our data were further analyzed by comparing the expression levels of specific genes between each treatment group and the control group. The volcano plots shown in Fig 1 represent the relative expression of individual genes plotted for each treatment group when compared to the control group, with the x-axis being the log_2_ (fold change) and the y-axis being the log_10_ (*p*-value). The black dots represent individual genes with expression levels that were not significantly different between the two groups (*p* > 0.01 and/or log_2_ (fold change) > 1.5). The red dots indicate genes with significantly different expression levels between the treatment and the control group (*p* < 0.01 and log_2_ (fold change) > 1.5). The green dots represent genes that only met the cutoff value for the fold-change (log_2_ (fold change) ≥ 1.5). The blue dots represent genes that only met the cutoff for the *p*-value (*p* > 0.01). Lastly, the red dots on the left half of the x-axis indicate genes that were down-regulated, whereas the red dots on the right half of the x-axis indicate individual genes that were up-regulated. When comparing the volcano plots, we found that most of the differentially expressed genes between the control group and the FC group, as well as those between the control group and the CC group, were up-regulated (Fig 1A and 1B, respectively), while most differentially expressed genes between the control group and A group were down-regulated (Fig 1C).

When we focused our analysis only on genes that were up-regulated across treatment groups, we found that six gene products overlapped between the FC and CC treatment groups compared to the controls. Those were “menin” (GB44079), “chymotrypsin inhibitor” (GB52203), “metabotropic glutamate receptor B (Glurb)” (GB53857), “spermatogenesis-defective protein 39 homolog” (GB42785), “zinc finger protein 423-like” (GB41288), and “unknown” (GB47207). One gene product that was up-regulated in the amitraz-laden wax vs. the control group comparison, “spermatogenesis-defective protein 39 homolog” (GB42785), was also up-regulated in the FC and CC treatment groups compared to the control.

**Fig 2:**
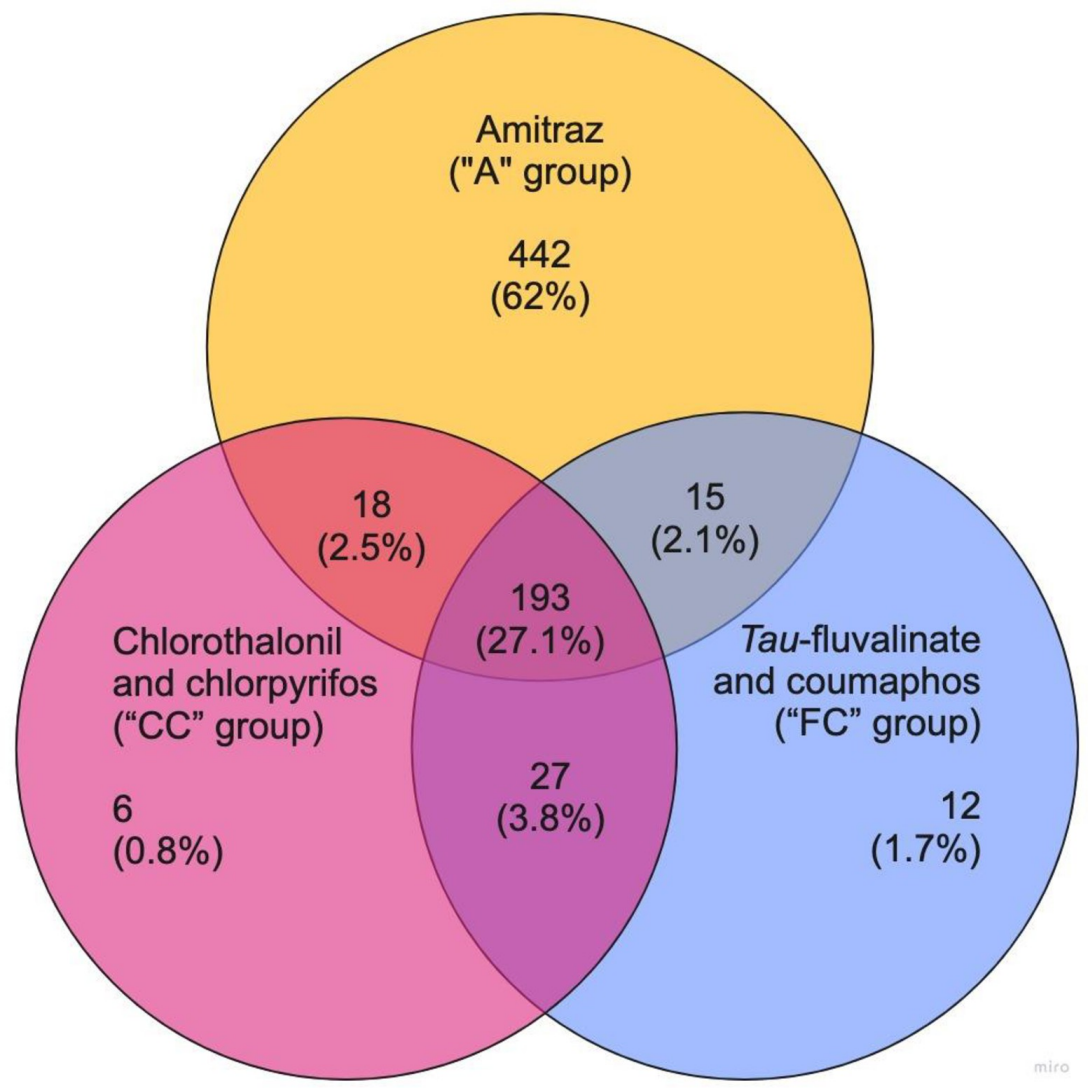
Venn diagram of differentially expressed genes (“DEGs”) across treatment groups. The number (and percentage) inside each circle represents the number of DEGs between the different comparisons (control group versus each treatment group). Each color represents a different comparison: 1) Yellow is amitraz only (“A” group), magenta is chlorothalonil and chlorpyrifos (“CC” group), and 3) blue is *tau*-fluvalinate and coumaphos (“FC” group). Only annotated genes were considered in these comparisons. The overlapping numbers represent the mutual differentially expressed genes between the different comparisons, while the non-overlapping numbers represent the genes that were unique to each condition. Venn diagram created with the online application Miro.

We also created a Venn diagram to display the number of unique genes that were expressed between all treatment groups (Fig 2). We found 12 unique DEGs in the FC treatment group when compared to the CC, A, and control groups. Of these, four were down-regulated and eight were up-regulated. A description of the 12 genes is presented in S5 Table. We also found six unique DEGs in the CC treatment group when compared to the FC, A, and control groups. Three of those genes were up-regulated and three were down-regulated. The descriptive list of all six genes is given in S6 Table. Finally, a much higher number of DEGs, 442, was unique in the A treatment group when compared to the FC, CC, and control groups. Of those, 35 were up-regulated and 407 were down-regulated. A complete list of those genes is given in S7 Table.

When examining unique DEGs only between pairs of treatment groups, we found 27 DEGs between the FC and CC treatment groups (Fig 2). One gene was down-regulated, and 26 genes were up-regulated (S8 Table). Likewise, there were 15 unique DEGs, one that was down-regulated, and 14 that were up-regulated, when comparing the FC treatment group to the A treatment group (S9 Table). Finally, there were 18 unique DEGs, two that were down-regulated and 16 that were up-regulated, when comparing the CC treatment group to the A treatment group (S10 Table). Overall, there were 193 DEGs that all treatment groups had in common, all of which were up-regulated (S11 Table).

### 3.2. Functional clustering analysis of differentially expressed genes

**Fig 3:**
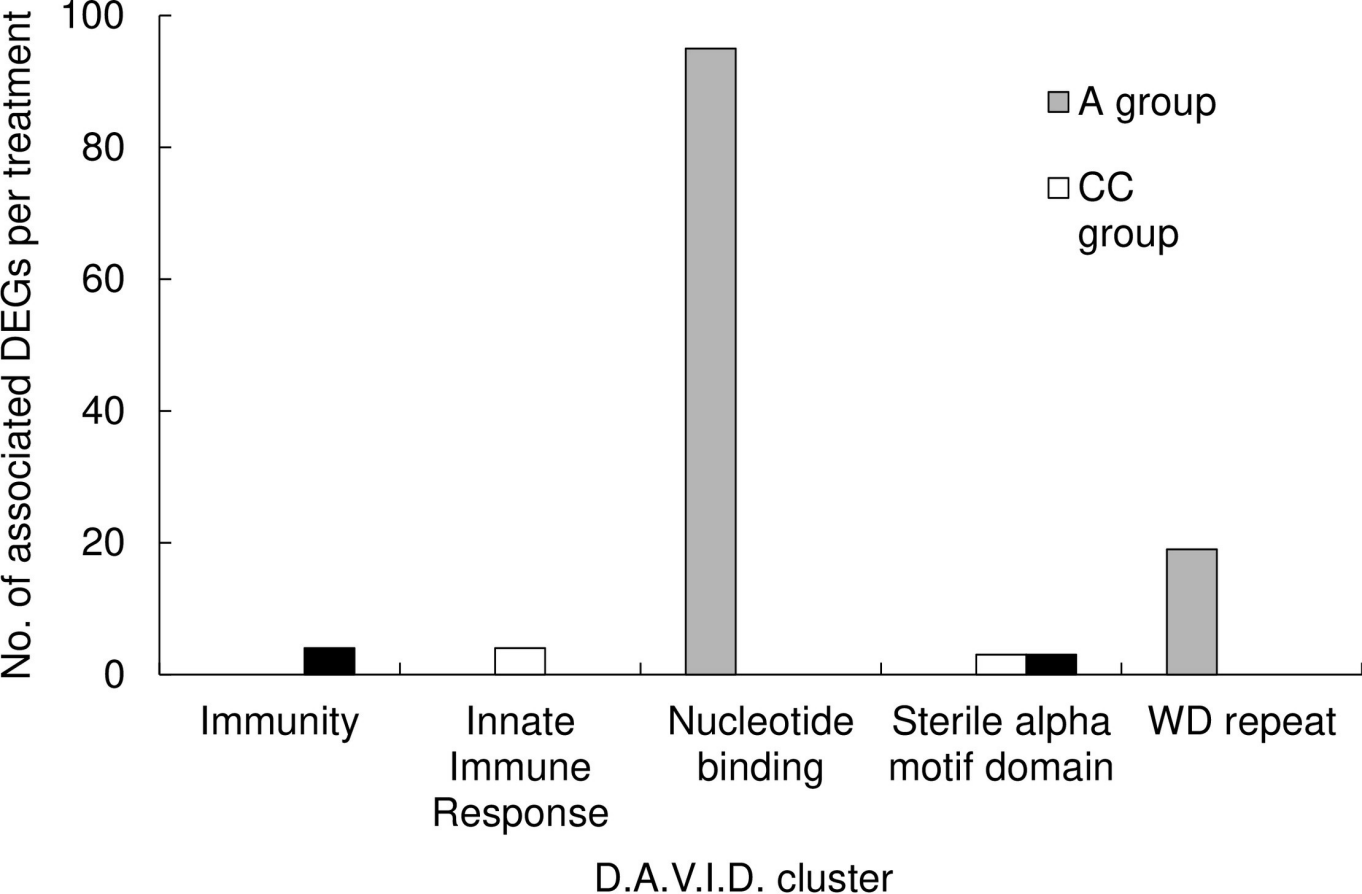
D.A.V.I.D. functional clustering analysis groupings with the number of associated differentially expressed genes (DEGs) for each treatment group compared to the untreated control group. For a list of all the genes per cluster see Tables 4–6. FC group = wax containing a combination of *tau*-fluvalinate and coumaphos; CC group = wax containing a combination of chlorpyrifos and chlorothalonil; and A group = wax containing amitraz.

Gene lists for all up- and down-regulated genes in the transcriptomes of brain tissues from queens that were reared either in untreated wax (control), or in wax containing either a mix of *tau*-fluvalinate and coumaphos, a mix of chlorpyrifos and chlorothalonil, or amitraz alone, were compiled into six different lists for D.A.V.I.D. functional annotation clustering analysis (Fig 3, Tables 4–6 and S12–S14 Tables). Of the 241 genes that were up-regulated in the *tau*-fluvalinate and coumaphos group, 204 were identified in the D.A.V.I.D. database (Table 4). From these, there were two clusters identified; four genes belonged to the “Immunity” cluster with an enrichment score of 1.99, and three genes belonged to the “sterile alpha motif domain” with an enrichment score of 1.19 (see S12 Table for protein descriptions). No clusters were identified in the genes that were down-regulated in the FC group. Furthermore, of the of 238 genes that were up-regulated in brain tissue from queens reared in wax mixed with chlorpyrifos and chlorothalonil, 201 were found in the D.A.V.I.D. database (Table 5), and two clusters were identified; four genes belonged to the “innate immune response” category with an enrichment score of 2.06, and three genes belong to the “sterile alpha motif domain” with an enrichment score of 1.18 (see S13 Table for protein descriptions). No clusters were identified in the genes that were down-regulated. Finally, of the 617 genes that were down-regulated in brain tissue from queens that were reared in amitraz-laden wax, 543 were found in the D.A.V.I.D. database (Table 6). From these, there were two clusters identified; 95 genes belonged to the “nucleotide-binding” category with an enrichment score of 1.99, and 19 genes belonged to the “WD repeat” category with an enrichment score of 1.19 (see S14 Table for protein descriptions). No clusters were identified in the list of genes that were up-regulated in the amitraz group. Finally, we took the 193 up-regulated DEGs that all the treatment groups had in common and conducted the D.A.V.I.D. functional annotation clustering analysis. Two clusters were identified; 27 genes belonged to the “nucleotide-binding” category with an enrichment score of 2.87, and 16 genes belonged to the “WD40/YVTN repeat-like-containing domain” with an enrichment score of 1.93 (Fig 3 and S15 Table).

**Table 4:** Cluster analysis of differentially expressed genes in brain tissue from queens reared in wax containing a mix of *tau*-fluvalinate and coumaphos ("FC") compared to those reared in untreated (control) wax, using the online tool D.A.V.I.D. (Huang et al. 2009).

Annotation	FDR	Gene list
***Genes up-regulated in brains from queens reared in FC-laden wax vs*. *those in the control group***		
***Cluster 1*: *Enrichment Score = 1*.*99***		
Immunity	1.35E-03	GB51223, GB47546, GB51306, GB47805
Innate immunity	1.35E-03	GB51223, GB47546, GB51306, GB47805
GO:0045087~innate immune response	1.42E-03	GB51223, GB47546, GB51306, GB47805
Antibiotic	1.21E-02	GB51223, GB47546, GB51306
Antimicrobial	1.21E-02	GB51223, GB47546, GB51306
GO:0042742~defense response to bacterium	1.51E-02	GB51223, GB47546, GB51306
Cleavage on pair of basic residues	2.88E-02	GB51223, GB47546, GB51306
***Cluster 2*: *Enrichment Score = 1*.*19***		
IPR001660:Sterile alpha motif domain	4.97E-02	GB49355, GB41982, GB54320

**Table 5:** Cluster analysis of differentially expressed genes in brain tissues from queens reared in wax containing a mix of chlorpyrifos and chlorothalonil ("CC") compared to those reared in untreated (control) wax, using the online tool D.A.V.I.D. (Huang et al. 2009).

Annotation	FDR	Gene list
***Genes up-regulated in brains from queens reared in CC-laden wax vs*. *those in the control group***		
***Cluster 1*: *Enrichment Score = 2*.*06***		
GO:0045087~innate immune response	9.72E-04	GB51223, GB47546, GB51306, GB47805
Immunity	1.26E-03	GB51223, GB47546, GB51306, GB47805
Innate immunity	2.78E-02	GB51223, GB47546, GB51306, GB47805
Antibiotic	1.16E-02	GB51223, GB47546, GB51306
Antimicrobial	1.16E-02	GB51223, GB47546, GB51306
GO:0042742~defense response to bacterium	1.18E-02	GB51223, GB47546, GB51306
Cleavage on pair of basic residues	2.75E-02	GB51223, GB47546, GB51306
***Cluster 2*: *Enrichment Score = 1*.*18***		
IPR001660:Sterile alpha motif domain	4.97E-02	GB49355, GB41982, GB54320

**Table 6:** Cluster analysis of differentially expressed genes in brain tissues from queens reared in wax containing amitraz ("A") compared to those reared in untreated (control) wax, using the online tool D.A.V.I.D. (Huang et al. 2009).

Annotation	FDR	Gene list
***Genes up-regulated in brains from queens reared in amitraz-laden wax vs*. *those in the control group***		
***Cluster 1*: *Enrichment Score = 1*.*99***		
Nucleotide-binding	4.05E-03	GB48362, GB53173, GB55431, GB46007, GB40217, GB42814, GB54281, GB41745, GB41358, GB41038, GB45876, GB42664, GB43553, GB50415, GB49490, GB50297, GB50274, GB54498, GB50652, GB47422, GB54593, GB46039, GB50193, GB44515, GB44536, GB44534, GB44237, GB43222, GB42297, GB44131, GB45284, GB48362
ATP-binding	9.79E-03	GB47573, GB53173, GB55431, GB46007, GB40217, GB42814, GB41745, GB41038, GB45876, GB42664, GB43553, GB50415, GB49490, GB50297, GB50274, GB50652, GB47422, GB50193, GB44515, GB44534, GB43222, GB42297, GB44131, GB45284, GB48362
GO:0005524~ATP binding	2.78E-02	GB47573, GB53414, GB47232, GB53036, GB53173, GB55431, GB46007, GB46569, GB45932, GB40217, GB42814, GB41745, GB46705, GB44305, GB41038, GB45876, GB42664, GB43553, GB50415, GB54938, GB49490, GB49485, GB50297, GB50274, GB50511, GB53320, GB50652, GB47422, GB47269, GB55144, GB49507, GB50193, GB44515, GB45107, GB44534, GB43222, GB42297, GB44131, GB45284
***Cluster 2*: *Enrichment Score = 1*.*19***		
WD repeat	2.12E-02	GB46242, GB44523, GB40271, GB50459, GB41547, GB42945
IPR015943:WD40/YVTN repeat-like-containing domain	5.02E-02	GB51542, GB46242, GB52153, GB45923, GB41547, GB42945, GB44538, GB43438, GB44523, GB41520, GB44685, GB40271, GB50459, GB54805

### 3.3. Real time quantitative PCR (RT-qPCR) for confirmation of RNA sequencing data

**Fig 4:**
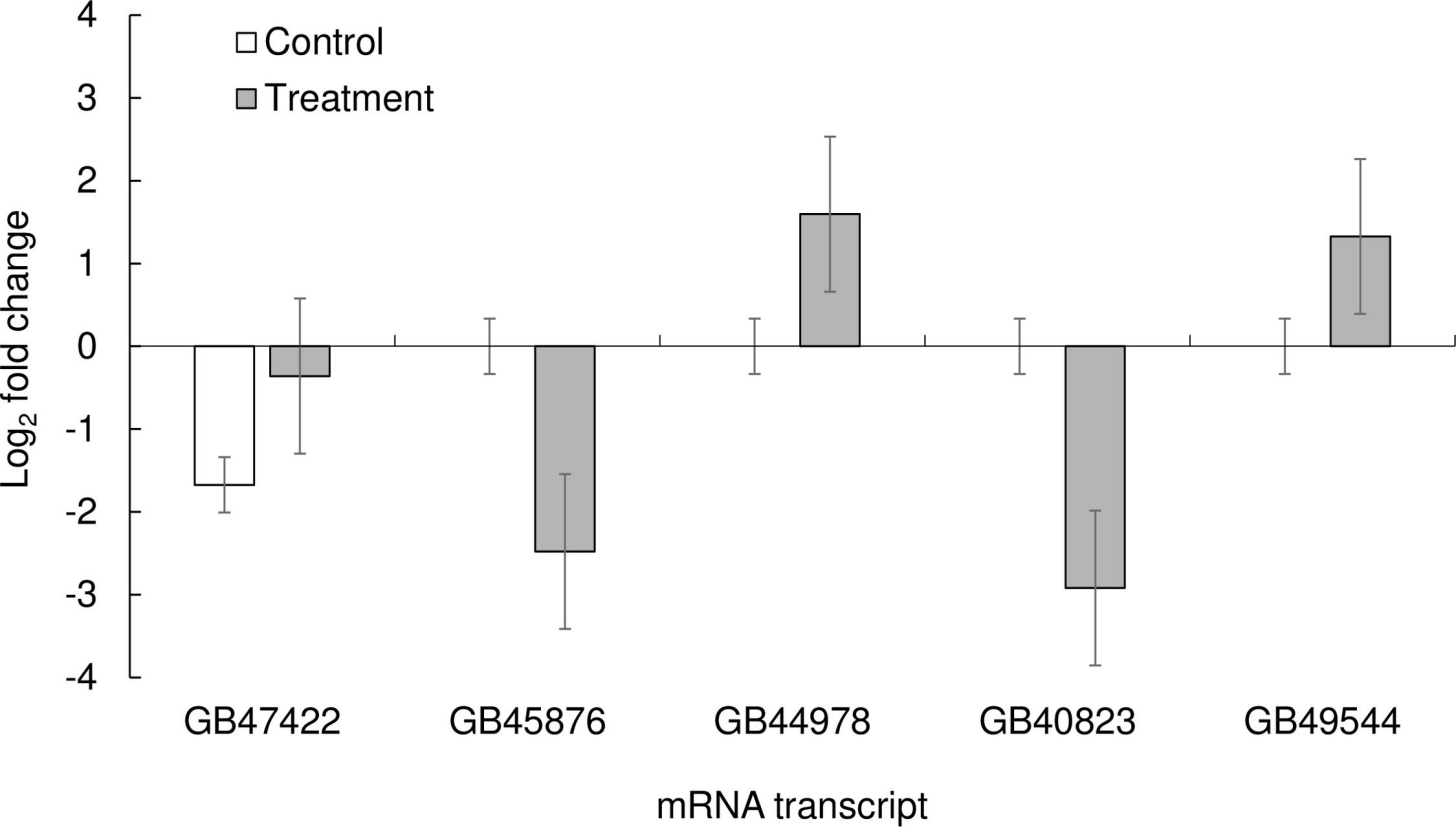
Log_2_ fold change of the relative expression of five individual genes when comparing the expression of each gene in the brain tissue from control queens (those that were reared in pesticide-free wax) relative to the expression of the housekeeping gene AF441189 (*Apis mellifera* ribosomal protein 49) (white bars), or the expression of each gene in the brain tissue from queens that were reared in amitraz-laden wax relative to the expression AF441189. Log_2_ fold change values > 0 denote up-regulation, while values < 0 denote down-regulation. The BeeBase gene identifier was used for the Gene ID (Munoz-Torres et al. 2010).

Five transcripts were selected for RT-qPCR analysis from our RNA sequencing data. The mRNAs were GB40823 (Vitellogenin receptor), GB49544 (Vitellogenin), GB47422 (Microtubule-associated serine/threonine-protein kinase 3), and GB45876 (Ribosomal protein S6 kinase beta-1-like), and they were selected because they are all involved in egg production by queens. GB44978 (Chitinase domain-containing protein 1) was also chosen because the other five mRNA targets were down-regulated, and we needed one for confirmation of up-regulation. Contrary to our expectations, however, there was no statistical difference in expression between the endogenous control gene (AF441189) and the genes GB474422 (F-ratio_1,5_ = 4.16, *p* = 0.09), GB49544 (F-ratio_1,8_ = 3.41, *p* = 0.10), GB45876 (F-ratio = 1.07, *p* = 0.33), GB44978 (F-ratio_1,5_ = 3.66, *p* = 0.11), or GB40823 (F-ratio_1,6_ = 1.22, *p* = 0.31) (Fig 4).

## 4. Discussion

In this study, we analyzed the brain transcriptome of honey bee queens that were reared in beeswax containing field-relevant concentrations of pesticides that are commonly found in honey bee colonies. We did this in order to provide further insight about the role that pesticides play as developmental stressors of queens. Our results showed that the use of pesticide-laden wax indeed affects the queen’s brain transcriptome. In particular, brain tissue from queens reared in amitraz-laden wax had the highest number of DEGs compared to brain tissue from queens reared in wax containing either a combination of *tau*-fluvalinate and coumaphos, or a combination of chlorpyrifos and chlorothalonil. We should note, however, that by only using three biological replicates per tissue type for transcriptomic analysis, we may have underestimated the number of genes that could have been differentially expressed due to the pesticide treatments [82, 83]. Thus, a larger number of replicates could have yielded a higher number of DEGs caused by pesticide exposure. Despite this potential shortcoming, it is clear that exposure to pesticide-laden wax during development caused drastic up- and down-regulation of hundreds of genes in the queen’s brain.

Interestingly, brain tissue from queens belonging to the A group had very different gene expression profiles compared to the FC and CC groups. Compared to the control group, most of the DEGs in the A group were down-regulated, whereas most of the DEGs in the FC and CC groups were up-regulated. This could potentially be explained by the down-regulation of transcription and/or translation factors caused by amitraz. For example, amitraz caused the down-regulation of “transcriptional repressor CTCFL-like,” “elongation factor 1-alpha F2” (EF1a-F2), “transcription factor Maf,” “transcription initiation factor TFIID subunit 4-like,” and “elongation factor-like GTPase 1,” whose functions (as inferred from the model organism *D*. *melanogaster*) may be related to either transcription or translation [84–86]. Conversely, genes such as “putative transcription factor capicua,” “elongation factor-like GTPase 1,” and “transcription factor mblk-1-like” (Mblk-1), were up-regulated in the FC and CC groups. When inferring these genes’ function from *D*. *melanogaster* analyses, it appears that *tau*-fluvalinate and coumaphos, as well as chlorothalonil and chlorpyrifos, could potentially cause higher transcriptional or translational responses in the honey bee queen’s brain [85, 87].

Several studies have previously shown that *tau*-fluvalinate, coumaphos, and amitraz, all of which are used to control *Varroa*, persist and accumulate in the beeswax matrix over time [9, 11, 24, 27, 71, 88, 89]. Most other studies have indicated that the presence of these products in the hive causes some detrimental off-target effects to honey bees. For example, exposure to *tau*-fluvalinate causes queens to have lower body weight [53]. It can also causes them to die within the first six months after they are placed into new colonies [90, 91] or to get superseded at high rates [91]. Likewise, exposure to coumaphos during development can cause lower ovary and body weight [53], as well as lower survival [54, 92].

Most recently, work in our laboratory showed that exposure of queen larvae during development to wax containing either a combination of *tau*-fluvalinate and coumaphos, or with amitraz alone, affected many aspects of worker behavior and queen physiology [25, 26, 72]. For example, we found that queens exposed to amitraz during development exhibited higher mating frequency than queens reared in pesticide-free beeswax [25]. Furthermore, Walsh et al. [26] found that rearing queens in pesticide-laden wax caused significantly lower attraction of queen tending workers to the contents of the queens’ mandibular glands. Those differences likely occurred because there were significant differences in the chemical composition of the glands’ contents. However, it is important to note that not all of the traits we measured were affected by pesticide exposure. For example, we did not find differences in body size of either virgin or mated queens, and sperm viability in the spermathecae of mated queens did not differ between control queens and those reared in pesticide-laden wax [72]. Nevertheless, and in direct relation to the current study (for reasons explained below), exposure to pesticide-laden wax during development caused mated queens to have smaller worker retinues and lower egg-laying rates than queens reared in pesticide-free wax [26].

Interestingly, we observed lower expression of farnesol dehydrogenase in brain tissue from queens raised in the amitraz group compared to those in the control group. This gene is associated with juvenile hormone (JH) production by the corpora allata, which is located in the brain [93]. It is well established that JH is an inducer of ovarian development at the beginning of the queen’s larval phase [94, 95]. Furthermore, high levels of JH during the last larval instar of queen development inhibit apoptosis in the ovarian germ cells, which helps to preserve the development of ovarian filaments and thus, increased queen fecundity [96–99]. Clearly, the roll of JH is different in adults than in developing larvae [100]. Because the brain is the primary production site of JH, and we observed lower expression of farnesol dehydrogenase (which is involved in the production of JH), we propose that the lower expression of farnesol dehydrogenase in the brain could have downstream effects in the expression of JH in other queen body tissues. However, while farnesol dehydrogenase was down-regulated by amitraz in our study, Walsh et al. [72] did not observe a difference in ovariole number for queens reared in amitraz-laden wax compared to control queens. Thus, the down-regulation of farnesol dehydrogenase by our amitraz treatment may not to be sufficient to cause a decrease in ovariole number in queens, as perhaps the expression of other genes in the JH production pathway might be sufficient for proper ovariole development. In addition, it would be interesting to measure the levels of JH in the hemolymph to determine if these levels might be lower in queens reared in amitraz-laden wax compared to control queens.

Amitraz has been the most widely used miticide to control for *Varroa* mites for the last 20 years, even though some studies have documented mite populations that are now resistant to amitraz in the U.S. [34, 31], Argentina [101], Czechia [102], and France [103]. Given the importance of amitraz as the main “go-to” miticide in U.S. commercial beekeeping operations, more studies looking at the non-target effects of amitraz exposure on honey bees are needed.

Amitraz is a formamidine that targets tyramine and octopamine receptors in insects [104–106]. We chose to focus on the DEGs in the brain tissue of queens reared in wax contaminated with amitraz partly because our previous work showed that queens reared in amitraz-laden wax laid significantly fewer eggs than queens in any other treatment group [26] We therefore aimed to find DEGs in queens from the amitraz group that were involved in egg production and identified five genes of interest: GB40823 (Vitellogenin receptor), GB49544 (Vitellogenin), GB50425 (Maternal gene required for meiosis), GB47422 (Microtubule-associated serine/threonine-protein kinase 3), and GB45876 (Ribosomal protein S6 kinase beta-1-like) (Fig 4). Interestingly, the transcriptomic analysis revealed that all five of these genes were down-regulated in brain tissue from queens reared in amitraz-laden wax compared to the controls. We should note, however, that our gene confirmation results did not align with the results from the transcriptomic analysis: while all five of the genes should have been down-regulated, while the positive control gene, GB44978, should have been up-regulated in the RT-qPCR analysis, we did not find that pattern to hold in the confirmation analysis. However, this is a common occurrence in some transcriptome studies, given that sometimes the genes that are used in the qPCR confirmation analysis actually end up being expressed in the opposite direction that was obtained in the transcriptomic analysis [107].

Vitellogenin (Vg) and vitellogenin receptor are involved in the production of the egg yolk protein necessary for oogenesis, as well as increased worker and queen longevity [108–110]. Microtubule-associated serine/threonine-protein kinase 3 is a member of the kinase gene family that is generally responsible for organelle and vesicle transport and segregation of chromosomes during mitosis [111, 112]. Finally, Ribosomal protein S6 kinase beta-1-like is activated by royalactin, which increases the activity of mitogen, and then activates juvenile hormone, an essential hormone for ovary development [113]. Interestingly, a recent study showed that amitraz causes an increase honey bee cardiac function and decrease survival of bees highly infected with viruses [42]. However, we do not know the role that these genes play in the brain, or whether differential expression in the brain causes downstream effects in expression in other tissues. These results provide the first evidence that amitraz contamination of wax can impair the proper expression of genes necessary for egg production, which in turn likely causes downstream physiological changes that lower a queen’s ability to lay eggs at full capacity.

Our study also showed differential gene expression, mostly up-regulation, in brain tissue from queens that were reared in wax containing either a combination of *tau*-fluvalinate and coumaphos, or a combination of chlorothalonil and chlorpyrifos. Previous work in our laboratory showed that queens in these treatment groups attracted fewer workers around their retinue and laid significantly fewer eggs than queens in the control group [26]. While other studies have shown that these pesticides cause sub-lethal health effects to honey bee workers, our studies are unique in that they have focused on queens specifically. For example, exposure to *tau*-fluvalinate has been linked to other serious physiological issues, such as worker susceptibility to viral infections [114]. Exposure to coumaphos has also been shown to alter the expression of genes associated with immunity, development, and detoxification [37]. Furthermore, chlorpyrifos and chlorothalonil have been shown to cause indirect effects on honey bees including impairment of the retrieval of olfactory memories, as well as negative synergistic effects in combination with other pesticides [115, 116]. In addition, both chlorpyrifos and chlorothalonil have been shown to decrease the lifespan and overall health of developing workers [117]. However, those studies were all conducted on workers, leaving a gap in knowledge on queen responses to pesticide exposure to these common pesticides. If such effects on workers are similar to those on queens, one could speculate that exposure to chlorpyrifos and chlorothalonil during development may result in shorter queen lifespan.

Even though honey bees (like other insects) lack adaptive immunity, they have evolved a highly effective innate immune system that enables them to reduce the impact of biotic and environmental stressors through behavioral, cellular, and humoral responses [71, 109–120]. In particular, honey bees produce antimicrobial peptides (AMPs), including *abaecin* and *hymenoptaecin*, through three signaling pathways (Toll, Imd, and Jak/STAT) in response to pathogens and parasites [121]. Our functional clustering analysis showed that brain tissue from queens reared in wax containing either *tau*-fluvalinate and coumaphos, or chlorothalonil and chlorpyrifos, showed highly clustered genes related to “Innate immune response.” Furthermore, *hymenoptaecin* and *apidaecin* were up-regulated in the queen’s brain after exposure to *tau*-fluvalinate and coumaphos during development. Previous work has revealed conflicting results as to whether *tau*-fluvalinate and coumaphos affect the honey bee’s immune system. For example, two studies showed no differences in the expression of genes related to immunity after workers were exposed to *tau*-fluvalinate or coumaphos compared to unexposed workers [71, 122], whereas other studies have shown that glucose oxidation activity, a measure of social immunity, is increased after exposure to *tau*-fluvalinate, coumaphos, and chlorothalonil [123, 124]. Another study showed that chlorothalonil acts synergistically with miticides to increase toxicity to honey bee larvae [40]. While those studies were focused on workers and not queens, it is possible that the up-regulation of immune genes in our study could be a stress response from pesticide exposure, not due to a pathogen or a parasite. For instance, Mckenna et al. [125] recently found that *Lucilia sericata* blow flies exposed to the ESKAPE bacterial pathogen *Acinetobacter baumannii* exhibit microbe-specific induction of immune genes, while the same genes were differentially expressed in *Drosophila sechellia* when feeding on toxic octanoic acid [126].

Several studies have shown that colonies in commercial beekeeping operations are contaminated with miticides and other agrochemicals, often at high levels [9, 11]. It is not surprising, therefore, that rearing queens in contaminated wax affects worker behavior and queen reproductive physiology. Our study reveals new data showing that exposure to these pesticides during development also provokes changes in the mated queen’s brain transcriptome. Because the queen is the sole reproductive head of a honey bee colony [59], mitigating the stressors that compromise queen health has become an increasingly important field of study. However, it is difficult to remedy the non-target effects to queens that are caused by pesticides, particularly miticides. This is because the beekeeping industry still relies heavily on the use of miticides to control the *Varroa* mite. While ceasing the use of miticides is unrealistic, beekeepers should practice better integrated pest and pollinator management (IPPM) techniques whenever possible to reduce their sole reliance on pesticides for *Varroa* control. Such IPPM methods include the replacement of old combs, the use *Varroa*-tolerant genetic stocks, the careful selection of apiary locations to reduce *Varroa* drift, the trapping of pupating drones (and thus mites), and the manual break in the brood cycle, among other practices [127]. While these methods are often inefficient based on time and costs, especially for commercial beekeepers, our studies and those of others [128] continue to reveal troubling effects of residual pesticide exposure during honey bee development and its effects on bees in the adult stage. Further research should continue to investigate these questions to fully understand how pesticide accumulation in the beeswax matrix affects the development and overall health of honey bee workers, as well as queens.

## Supporting information

S1 Table Sequencing statistics for 48 cDNA libraries created. Each library’s identification number (ID #) was labeled as follows: sequential number, followed by the treatment group, and ending in the individual brain tissue replicate (A, B, or C) that was sequenced. The treatment groups correspond to brain tissue from queens that were reared either in untreated (control) wax (“C”), wax mixed with amitraz (“A”), wax mixed with a combination of chlorothalonil and chlorpyrifos (“CC”), or wax mixed with *tau*-fluvinate and coumaphos (“FC”). The “total number of reads mapped” is the number of reads that were mapped to the honey bee genome.
(XLSX)

S2 Table List of all differentially expressed genes (247 DEGs) from brain tissue of queens that were reared in wax mixed with a combination of *tau*-fluvalinate and coumaphos ("FC" group) compared to those reared in untreated (control) wax. The genes are ranked by their log_2_ fold change value (LFC). Genes are identified by their BeeBase identifer (Gene ID). The *p*-value is the indiviual value for each gene. FDR is the false discovery rate.
(XLSX)

S3 Table List of all differentially expressed genes (244 DEGs) from brain tissue of queens that were reared in wax mixed with a combination of chlorpyrifos and chlorothalonil ("CC" group) compared to those reared in untreated (control) wax. The genes are ranked by their log_2_ fold change value (LFC). Genes are identified by their BeeBase identifer (Gene ID). The *p*-value is the indiviual value for each gene. FDR is the false discovery rate.
(XLSX)

S4 Table List of all differentially expressed genes (668 DEGs) from brain tissue of queens that were reared in wax mixed with a combination of amitraz ("A" group) compared to those reared in untreated (control) wax. The genes are ranked by their log_2_ fold change value (LFC). Genes are identified by their BeeBase identifer (Gene ID). The *p*-value is the indiviual value for each gene. FDR is the false discovery rate.
(XLSX)

S5 Table List of unique differentially expressed genes in the brain tissue of queens reared in wax containing a mix of *tau*-fluvalinate and coumaphos, as compared to those reared in either untreated (control) wax, or wax containing a mix of chlorthalonil and chlorpyrifos, or wax containing only amitraz. The genes are ranked by their log_2_ fold change (LFC). Gene are identified by their BeeBase identifer (Gene ID). The *p*-value is the indiviual value for each gene. FDR is the false discovery rate. See the Venn diagram in Fig 2 with all the unique genes that were expressed between all treatment groups.
(XLSX)

S6 Table List of unique differentially expressed genes in the brain tissue of queens reared in wax containing a mix of chlorthalonil and chlorpyrifos, as compared to those reared in either untreated (control) wax, or wax containing a mix of *tau*-fluvalinate and coumaphos, or wax containing only amitraz. The genes are ranked by their log_2_ fold change (LFC). Gene are identified by their BeeBase identifer (Gene ID). The *p*-value is the indiviual value for each gene. FDR is the false discovery rate. See the Venn diagram in Fig 2 with all the unique genes that were expressed between all treatment groups.
(XLSX)

S7 Table List of unique differentially expressed genes in the brain tissue of queens reared in wax containing amitraz, as compared to those reared in either untreated (control) wax, or wax containing a mix of *tau*-fluvalinate and coumaphos, or a mix of chlorthalonil and chlorpyrifos. The genes are ranked by their log_2_ fold change (LFC). Gene are identified by their BeeBase identifer (Gene ID). The *p*-value is the indiviual value for each gene. FDR is the false discovery rate. See the Venn diagram in Fig 2 with all the unique genes that were expressed between all treatment groups.
(XLSX)

S8 Table List of unique differentially expresssed genes (27 DEGs) in the brain tissue of queens reared in wax containing a mix of *tau*-fluvalinate and coumaphos, as compared to those reared in wax containing a mix of chlorthalonil and chlorpyrifos. The genes are ranked by their log_2_ fold change (LFC). Gene are identified by their BeeBase identifer (Gene ID). The *p*-value is the individual value for each gene. FDR is the false discovery rate.
(XLSX)

S9 Table List of unique differentially expresssed genes (15 DEGs) in the brain tissue of queens reared in wax containing a mix of *tau*-fluvalinate and coumaphos, as compared to those reared in wax containing amitraz. The genes are ranked by their log_2_ fold change (LFC). Gene are identified by their BeeBase identifer (Gene ID). The *p*-value is the individual value for each gene. FDR is the false discovery rate.
(XLSX)

S10 Table List of unique differentially identifie genes (18 DEGs) in the brain tissue of queens reared in wax containing a mix of chlorothanoil and chlorpryrifos, as compared to those reared in wax containing amitraz. The genes are ranked by their log_2_ fold change (LFC). Gene are identified by their BeeBase identifier (Gene ID). The *p*-value is the individual value for each gene. FDR is the false discovery rate.
(XLSX)

S11 Table List of unique differentially expresssed genes (193 DEGs) that all treatment groups had in common. The genes are ranked by their log_2_ fold change (LFC). Gene are identified by their BeeBase identifer (Gene ID). The *p*-value is the indiviual value for each gene. FDR is the false discovery rate.
(XLSX)

S12 Table Functional annotation clustering of differentially expressed brain tissue between queens that were reared in untreated (control) wax and those reared in wax mixed with *tau*-fluvalinate and coumaphos ("FC") using the online tool D.A.V.I.D. (Huang et al. 2009). Recognized gene clusters were identified using the medium stringency of the software’s "Functional Annotation Clustering" settings. The reported genes are those that were clustered and had a false discovery rate (FDR) value < 0.05. Genes are reported using their BeeBase gene identifiers and their protein descriptions. See S2 Table for a complete list of all the genes that were differentially expressed in brain tissue between queens that were reared in untreated (control) wax and those reared in wax mixed with *tau*-fluvalinate and coumaphos.
(XLSX)

S13 Table Functional annotation clustering of differentially expressed brain tissue between queens that were reared in untreated (control) wax and those reared in wax mixed with chlorpyrifos and chlorothalonil ("CC") using the online tool D.A.V.I.D. (Huang et al. 2009). Recognized gene clusters were identified using the medium stringency of the software’s "Functional Annotation Clustering" settings. The reported genes are those that were clustered and had a false discovery rate (FDR) value < 0.05. Genes are reported using their BeeBase gene identifiers and their protein descriptions. See S3 Table for a complete list of all the genes that were differentially expressed in brain tissue between queens that were reared in untreated (control) wax and those reared in wax mixed with chlorpyrifos and chlorothalonil.
(XLSX)

S14 Table Functional annotation clustering of differentially expressed brain tissue between queens that were reared in untreated (control) wax and those reared in wax containing amitraz using the online tool D.A.V.I.D. (Huang et al. 2009). Recognized gene clusters were identified using the medium stringency of the software’s "Functional Annotation Clustering" settings. The reported genes are those that were clustered and had a false discovery rate (FDR) value < 0.05. Genes are reported using their BeeBase gene identifiers and their protein descriptions. See S4 Table for a complete list of all the genes that were differentially expressed in brain tissue between queens that were reared in untreated (control) wax and those reared in wax containing with amitraz.
(XLSX)

S15 Table Functional annotation clustering of all the differentially expressed genes that the three treatment groups (A, FC, and CC) had in common using the online tool D.A.V.I.D. (Huang et al. 2009). Recognized gene clusters were identified using the medium stringency of the software’s "Functional Annotation Clustering" settings. The reported genes are those that were clustered and had a false discovery rate (FDR) value < 0.05. Genes are reported using their BeeBase gene identifiers and their protein descriptions. See S11 Table for a complete list of all the genes that were shared between treatment groups.
(XLSX)
